# Pneumocephalus and empyema in the subarachnoid space: An unusual imaging feature secondary to spinal-epidural anesthesia

**DOI:** 10.1016/j.heliyon.2024.e26029

**Published:** 2024-02-10

**Authors:** Yan Liu, Zhen Li, Guohui Li, Xiaoping Tan

**Affiliations:** a Department of Neurology, Shengjing Hospital of China Medical University, Shenyang, China; b Department of Neurology, Yan'an People's Hospital, Yan'an, China; c Department of Neurosurgery, Yan'an People's Hospital, Yan'an, China

**Keywords:** Pneumocephalus, Empyema, Septic meningitis, Anesthesia, Spinal-epidural anesthesia

## Abstract

Pneumocephalus and empyema in the subarachnoid space secondary to spinal-epidural anesthesia are very rare and have not previously been reported, to our knowledge. Here, we describe the imaging features of an older woman presenting with pneumocephalus plus subarachnoid and intraventricular empyema due to Staphylococcus epidermidis infection after spinal-epidural anesthesia, with the aim of raising awareness regarding this serious complication.

## 1 Introduction

Pneumocephalus, defined as the presence of gas in the intracranial cavity, is frequently observed in neurosurgery, and after trauma or infection with gas-producing organisms; headache is the most frequent symptom [1]. Pneumocephalus is a well-known but rare complication of spinal or epidural anesthesia, and it has been reported in only a small subset of patients [[2], [3], [4]]. To our knowledge, pneumocephalus accompanied by empyema in the subarachnoid space as a complication of combined spinal-epidural anesthesia has not previously been reported. Here, we describe the imaging features of an older woman presenting with pneumocephalus plus subarachnoid empyema due to Staphylococcus epidermidis infection after spinal-epidural anesthesia, to increase awareness regarding this serious and rare complication.

## 2 Case Description

**Fig. 1:**
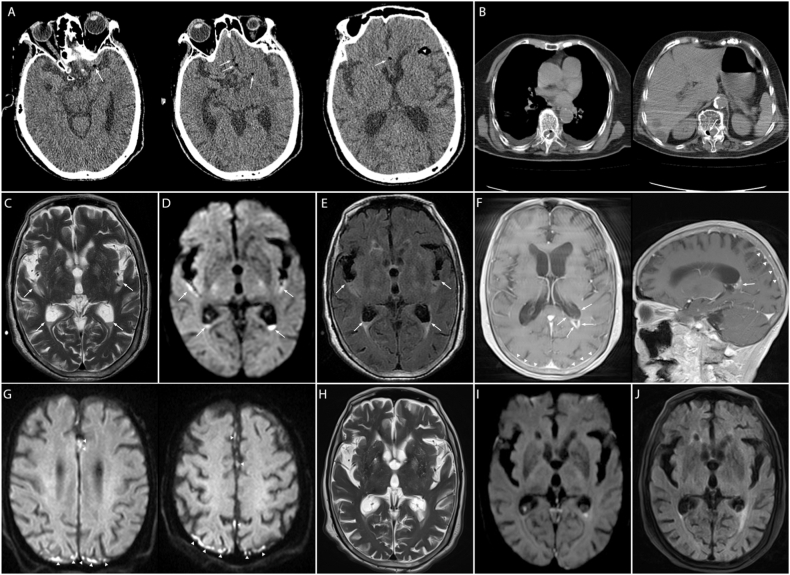
Brain computed tomography (CT) scan, showing pneumocephalus in the subarachnoid space (A). Gas density is present in the spinal canal on chest CT (B). Magnetic resonance imaging (MRI) findings, indicating empyema in the trigone of the lateral ventricle, the Sylvian fissure and the cerebral hemispheric sulcus (C–G), and significantly improved neuroimage changes after treatment (H–J).

A 92-year-old previously healthy woman with a left femoral neck fracture due to an accidental fall, and no prior history of brain injury or surgery or drug use, presented with high fever, nausea, vomiting and altered mental status for 4 hours, after combined spinal-epidural anesthesia for artificial femoral head replacement. At admission, an oral anticoagulant agent with rivaroxaban was prescribed to prevent lower limb venous thrombosis, on the basis of her high D-dimer value (1450 mg/mL, normal: 0–500). Her other laboratory test findings, including hemoglobin, white blood cell count, blood clotting function, and liver and kidney function, were within normal limits; however, postoperative blood tests indicated infectious leukocytosis. A brain CT scan performed 1 day after combined spinal-epidural anesthesia revealed typical pneumocephalus imaging features, including slight gas density in the suprasellar cisternae (Fig. 1A, arrows) and the anterior part of the Sylvian fissure (Fig. 1A, arrowheads). Pneumorrhachis was also noted on subsequent chest CT (Fig. 1B), thereby suggesting that the gas was likely to have originated from the difficult and repeated spinal-epidural puncture and the use of the loss of resistance to air pressure test.

**Fig. 2:**
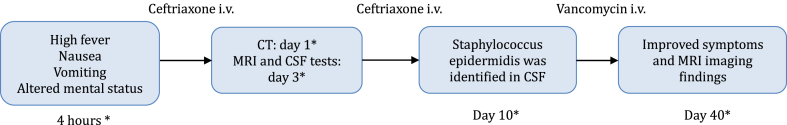
Flowchart of the clinical course of this case. *: Time interval after anesthesia CT: computed tomography MRI: magnetic resonance imaging CSF: cerebrospinal fluid.

On day 3 post-operatively, axial T2-weighted magnetic resonance imaging (MRI) indicated fluid level signs in the trigone of the bilateral lateral ventricle, the posterior part of the Sylvian fissure (Fig. 1C, arrows) and the cerebral hemispheric sulcus (Fig. 1C–G). Correspondingly, in these regions, we observed restricted diffusion on axial diffusion-weighted MRI (Fig. 1D, arrows), and increased fluid-attenuated inversion recovery MRI (FLAIR) hyperintense changes (Fig. 1E, arrows), thus suggesting empyema in the lateral ventricular and subarachnoid space. Postoperative empirical therapy with ceftriaxone was administered because of fever, and low molecular weight heparin was given for anticoagulation. On the subsequent MRI 10 days later, axial T1 postcontrast images showed remarkable linear enhancement of the subependymal region of lateral ventricles (Fig. 1F, arrows), parieto-occipital meninges and falx cerebri (Fig. 1F, arrowheads). In addition, restricted-diffusion patterns were observed in these lesion sites through diffusion-weighted MRI (Fig. 1G, arrowheads). Subsequently, cerebrospinal fluid (CSF) tests indicated increased moderately elevated intracranial pressure and inflammatory biochemical changes (CSF findings: 22219 leucocytes/μL; protein: 4.45 g/L; glucose: 0.29 mmol/L; chloride: 109.6 mmol/L). One week later, Staphylococcus epidermidis was identified in the etiologic workup of the CSF culture. Subsequently, the patient received antimicrobial treatment for 1 month with vancomycin, and her symptoms fully resolved. Follow-up MRI imaging at discharge indicated a disappearance of empyema in the Sylvian fissure and marked resolution in the lateral ventricles (Fig. 1H–J). A flowchart is shown in Fig. 2 to help readers follow this case.

## 3 Discussion

To our knowledge, pneumocephalus combined with empyema after spinal or epidural anesthesia has not previously been reported. The mechanisms of pneumocephalus in spinal or epidural anesthesia may be that a perfect conduit for air to enter the spinal canal can be created by removal of the core and syringe from the hollow epidural needle during the anesthesia operation [2]. This mechanism may be likely to occur in scenarios with difficult puncture, repeated negative pressure tests using loss of resistance to air technology and unsuccessful drainage of cerebrospinal fluid [5,6]. Unfortunately, these unexpected events—particularly repeated lumbar puncture, owing to a failure to obtain CSF, and negative pressure tests based on improper use of loss of resistance to air—also occurred in the anesthesia and might potentially explain the pneumocephalus in our case. In addition to the common causes of pneumocephalus, including open head trauma, neurosurgery, and intraspinal surgery, other rare causes have been reported, such as blunt chest trauma without spinal fractures [7] or noninvasive ventilation [8]. The formation of channels linking the skull cavity or spinal canal to the outside thus may provide a reasonable explanation for pneumocephalus in these rare cases, as observed in spinal or epidural anesthesia.

Furthermore, in certain conditions, such as non-strict sterilization of medical supplies or incomplete skin disinfection, bacteria such as Staphylococcus epidermidis and *Staphylococcus aureus* can enter the subarachnoid space and cause septic meningitis, which is also a rare complication of spinal or epidural anesthesia [9]. Additionally, a lack of surgical mask use is a frequent risk factor for septic meningitis associated with spinal, epidural and combined neuraxial anesthesia, and Streptococcus salivarius is the most prevalent bacterium in these cases [9]. In our case, we assumed that *Staphylococcus aureus* infection, originating from incomplete skin disinfection of the anesthetic manipulation area, was the direct cause of the intracranial empyema, and the difficult puncture increased the chances of this infection. Of note, infections with bacteria such as *Clostridium perfringens* [10] can also lead to pneumocephalus. However, in our case, *Staphylococcus aureus* was responsible for the intracranial empyema but not the pneumocephalus.

Consequently, we recommend vigilance in monitoring for infrequent complications of pneumocephalus and/or septic meningitis after spinal or epidural anesthesia. Clinicians should be aware of these rare characteristic imaging findings. Additionally, the following measures should be taken to avoid problems. First, the technical operation ability of spinal punctures should be improved, multiple puncture attempts should be avoided or decreased, and fine spinal needles should be used to avoid damage to the dura. Second, when the loss of resistance to air technique is used, air should not be injected into the epidural space when the resistance disappears. Alternatively, use of saline rather than air to identify the epidural space can avoid this complication [11]. Finally, standardized sterile technique is critical and minimizes the time required for spinal puncture.

## Informed Consent

We thank the patient's trustee for granting permission to the publication of all images, clinical data, and other data.

## Data availability statement

Data will be made available on request.

## CRediT authorship contribution statement

**Yan Liu:** Writing – review & editing, Formal analysis, Data curation, Conceptualization. **Zhen Li:** Writing – review & editing, Writing – original draft. **Guohui Li:** Writing – review & editing, Writing – original draft. **Xiaoping Tan:** Writing – review & editing, Formal analysis, Data curation, Conceptualization.

## Declaration of competing interest

The authors declare that they have no known competing financial interests or personal relationships that could have appeared to influence the work reported in this paper.
